# Genome-wide association studies of body size traits in Tibetan sheep

**DOI:** 10.1186/s12864-024-10633-3

**Published:** 2024-07-30

**Authors:** Dehui Liu, Xue Li, Lei Wang, Quanbang Pei, Jincai Zhao, De Sun, Qianben Ren, Dehong Tian, Buying Han, Hanjing Jiang, Wenkui Zhang, Song Wang, Fei Tian, Sijia Liu, Kai Zhao

**Affiliations:** 1 Qinghai Provincial Key Laboratory of Animal Ecological Genomics, Key Laboratory of Adaptation and Evolution of Plateau Biota, Northwest Institute of Plateau Biology, Chinese Academy of Sciences, No. 23 Xinning Road, Xining, Qinghai 810001 China; 2 University of Chinese Academy of Sciences, Beijing, 100049 China; 3 Qinghai Sheep Breeding and Promotion Service Center, Gangcha, Qinghai 812300 China; 4 Branch of Animal Husbandry and Veterinary of Heilongjiang Academy of Agricultural Sciences, Qiqihar, 161005 China; 5 Qinghai Livestock and Poultry Genetic Resources Protection and Utilization Center, Xining, 810000 China; 6 Animal Husbandry and Veterinary Station of Huzhu County of Qinghai Province, Huzhu, Qinghai 810500 China

**Keywords:** Tibetan sheep, Re-sequencing, GWAS, Body size traits, SNP

## Abstract

**Background:**

Elucidating the genetic variation underlying phenotypic diversity will facilitate improving production performance in livestock species. The Tibetan sheep breed in China holds significant historical importance, serving as a fundamental pillar of Qinghai’s animal husbandry sector. The Plateau-type Tibetan sheep, comprising 90% of the province’s population, are characterized by their tall stature and serve as the primary breed among Tibetan sheep. In contrast, Zhashijia sheep exhibit larger size and superior meat quality. These two species provide an excellent model for elucidating the genetic basis of body size variation. Therefore, this study aims to conduct a comprehensive genome-wide association study on these two Tibetan sheep breeds to identify single nucleotide polymorphism loci and regulatory genes that influence body size traits in Tibetan sheep.

**Result:**

In this study, the phenotypic traits of body weight, body length, body height, chest circumference, chest depth, chest width, waist angle width, and pipe circumference were evaluated in two Tibetan sheep breeds: Plateau-type sheep and Zhashijia Tibetan sheep. Whole genome sequencing generated 48,215,130 high-quality SNPs for genome-wide association study. Four methods were applied and identified 623 SNPs significantly associated with body size traits. The significantly associated single nucleotide polymorphisms identified in this study are located near or within 111 candidate genes. These genes exhibit enrichment in the cAMP and Rap1 signaling pathways, significantly affecting animal growth, and body size. Specifically, the following genes were associated: *ASAP1*, *CDK6*, *FRYL*, *NAV2*, *PTPRM*, *GPC6*, *PTPRG*, *KANK1*, *NTRK2* and *ADCY8*.

**Conclusion:**

By genome-wide association study, we identified 16 SNPs and 10 candidate genes associated with body size traits in Tibetan sheep, which hold potential for application in genomic selection breeding programs in sheep. Identifying these candidate genes will establish a solid foundation for applying molecular marker-assisted selection in sheep breeding and improve our understanding of body size control in farmed animals.

**Supplementary Information:**

The online version contains supplementary material available at 10.1186/s12864-024-10633-3.

## Background

Sheep are essential farm animals, with a wide distribution ranging from the Tibetan Plateau to the eastern regions of China. Based on their geographic distribution and genetic relationships, Chinese sheep can be classified into three primary lineages: Tibetan, Mongolian, and Kazakh [1]. Tibetan sheep (*Ovis aries*) exhibit robust adaptability to the alpine pastoral environment and harsh feeding and management conditions and maintain stable genetic properties [2]. As a crucial livestock species in pastoral regions, Tibetan sheep provide essential resources such as meat, milk, fur, and fuel for production and livelihoods, but also is intertwined with social and cultural aspects [3–6]. Due to the absence of systemic selection and breeding, Tibetan sheep showed evident limitations on production traits, such as a low reproductive rate, slow growth, and poor meat production. Zhashijia sheep are an ecological type of Tibetan sheep in Qinghai, which was formed through continuous crossbreeding between wild disc sheep and local sheep and years of natural selection and natural conditions. The Zhashijia family sheep is considered a plateau-type Tibetan sheep. Still, the two breeds differ significantly in body size, and Zhashijia is higher than the plateau type. Both of these two species are Tibetan sheep resources that have been developed through long-term breeding and natural confinement in the pastoral areas of Qinghai Province. However, they exhibit distinct differences in phenotypic characteristics and production traits. Therefore, elucidating the genetic basis underlying these phenotypic variations and identifying novel genes associated with essential characteristics will contribute to the enrichment of genetic resources for superior germplasm development and enhancement of growth performance in sheep.

Body size traits are widely recognized as crucial growth and health indicators, which are significantly influenced by feeding, management, and environmental factors [7, 8]. The noteworthy point is that body size exhibits moderate to high heritability [9–11]. Mature body size has been extensively investigated in humans, cattle, and other domestic animals. However, genomic bases underlying body size variation in sheep remain elusive [9, 12]. Since 1996, Risch et al. [13] recognized the limitations of conventional research methods, prompting the emergence of genome-wide association study as a novel approach. With advancements in high-density SNP microarrays, statistical methodologies, and complementary technologies, GWAS has been widely adopted in sheep genetic breeding programs [14–17]. Zhang [18] employed the SNP50K chip for genotyping 329 sheep from three breeds and identified *PFKFB4*, *TRHED*, *MEF2B*, *PLA2G6*, *RARB*, *OXSM*, *LRRC2*, *ADK*, and *SHISA9* candidate genes were significantly associated with pre-and post-weaning weight gain, weight at six months of age, chest circumference, and calf circumference. Subsequently, among these candidate genes, Zhang [19] further validated the *TRHED* and *MEF2B* genes [18] in Uzhumqin sheep. The study found a significant correlation between these two genes and weight and chest circumference at four months. Ghasemi [20] identified three significant SNPs on chromosome 1 associated with birth weight in 132 Lori-Bakhtiari sheep. These SNPs were predominantly located within the Member RAS Oncogene Family (*RAB6B*) and GRB10 interacting GYF protein 2 (*GIGYF2*) genes, suggesting their potential as candidate genes influencing sheep birth weight. In Baluchi sheep, GWAS revealed that five SNPs were significantly associated with the body weight of an eight-month-old lamb, and one SNP was located within the 50 Kb region near the *MTPN* gene that plays a crucial role in muscle growth and development [21]. Li [22] conducted GWAS on Tibetan sheep and 328 hybrid offspring, identifying ten candidate genes significantly associated with body size traits. These studies have yielded valuable genetic markers that can be utilized in sheep breeding.

Advancements in sequencing technology have propelled the advancement of genomics, with the sequencing of the sheep genome unveiling pivotal functional genes linked to traits such as meat quality, body size, and growth. Furthermore, it is feasible to pinpoint loci responsible for phenotypic differences caused by variations, which play a pivotal role in guiding the analysis of animal genetic mechanisms.

This study aimed to conduct a genome-wide association study (GWAS) using 11× whole genome resequencing data and body size traits of 193 sheep from plateau-type Tibetan and Zhashijia breeds. The objective was to identify candidate genes and SNP loci that influence body size traits in Tibetan sheep and to discover potential genetic markers for genomic selection in sheep breeding programs.

## Results

### Descriptive statistics

We conducted descriptive statistics and analyzed the phenotypic data of 193 Tibetan sheep. The results presented in Table 1 and Supplementary Table S1 include the mean, maximum, minimum, standard error, and coefficient of variation for body weight (BW: 40.4 kg), body length (BL: 66.28 cm), body height (BH: 69.66 cm), chest circumference (CC: 91.52 cm), chest depth (CD: 32.01 cm), chest width (CW: 21.52 cm), waist angle width (WW: 13.84 cm) and pipe circumference (PC: 7.38 cm). The coefficient of variation ranged from 6.86 to 21.73%, with the lowest and highest values observed for traits BH and BW, respectively. Finally, we performed correlation analyses for the eight phenotypic traits and found that only PC and WW were not significantly correlated, while all other phenotypic characteristics were significantly correlated (Supplementary Table S2).

**Table 1:** Descriptive statistics for the body size traits evaluated

Trait	Mean	Max	Min	SE	CV(%)
BW (kg)	40.04	77.1	21.9	0.63	21.73
BH (cm)	69.66	84	57	0.34	6.86
BL (cm)	66.28	80	41	0.39	8.12
CC (cm)	91.52	107.5	69.5	0.53	8.1
CW (cm)	21.52	30	17	0.15	9.67
CD (cm)	32.01	41	24	0.21	9.06
WW (cm)	13.84	16	10	0.08	7.86
PC (cm)	7.38	9.2	6.2	0.04	7.05

*Note* BW = body weight; BH = body height; BL = body length; CC = chest circumference; CW = chest width; CD = chest depth; WW = waist angle width; PC = pipe circumference

### Whole genome sequencing data

A total of 193 Tibetan sheep were subjected to whole genome sequencing, generating 5.49 Tb of raw reads with an average of 29.15 Gb per sample. The sequencing quality was high, with an average Q20 score of 97.15% and an average Q30 score of 91.15%. The GC content distribution in the 193 samples ranged from 41.65 to 45.78%, indicating no bias in library construction and sequencing (Supplementary Table S3). Compared to the reference genome, the average coverage depth ranged from 193 samples (Supplementary Table S4). Following SNP calling, we identified 48,755,632 SNP sites and classified them into 12 distinct mutation types. Among these mutations, C > T and G > A were the most prevalent mutations, occupying 19.5% and 19.6%, respectively, and A > T, C > G, G > C, and T > A were the most minor frequent mutations, occupying only 3.7% (Supplementary Fig. 1 and Table S5). Annotation results showed that 52.75% and 34.29% of SNPs were in intergenic and intron regions, and only 0.41% represented missense mutations (Supplementary Table S6).

### Population genetic diversity analysis

**Fig. 1:**
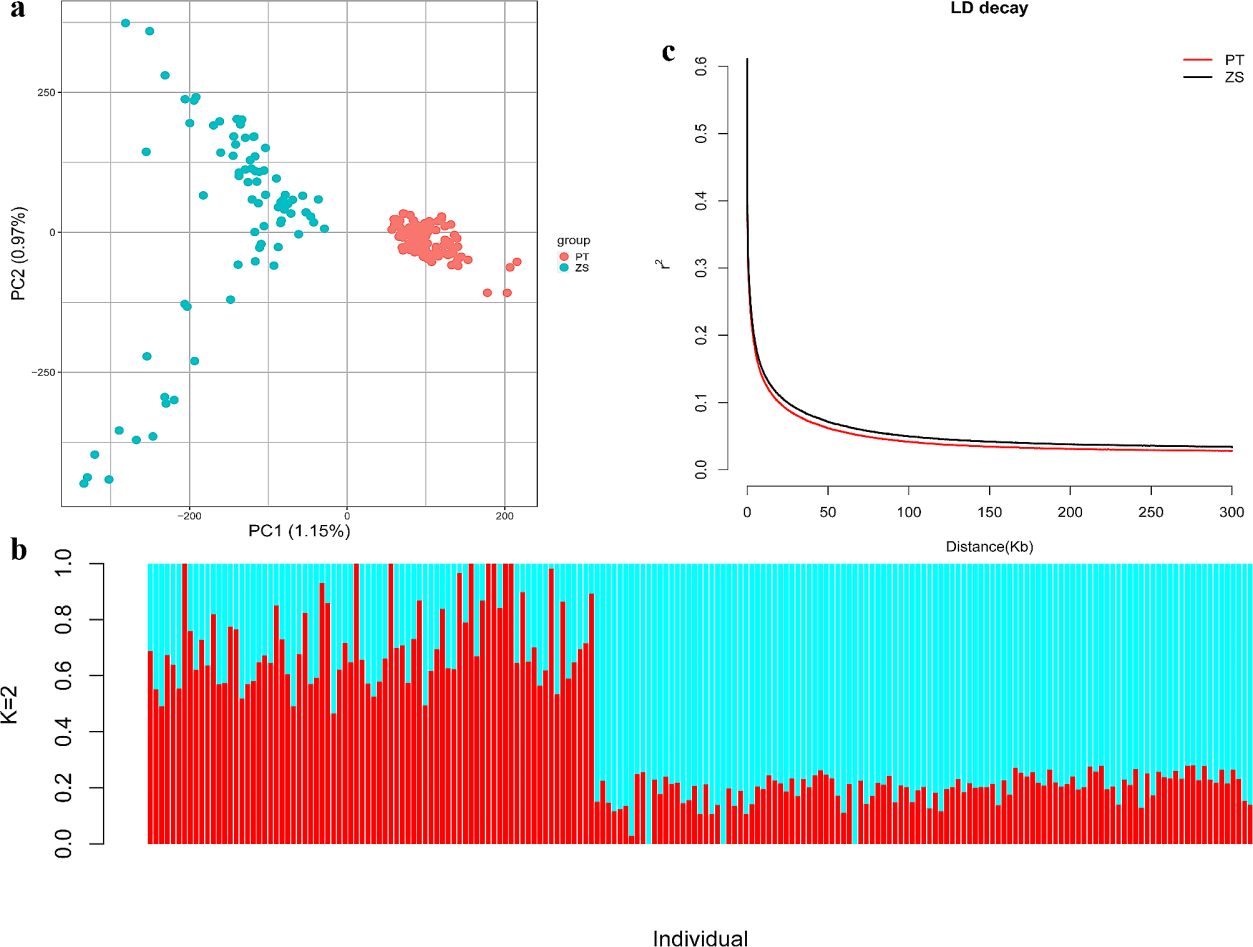
**a**: PCA analysis for the two populations of Tibetan sheep. PT indicates, and ZS denotes; **b**: Map of population genetic structure. The red color in the figure represents a subpopulation component, and the blue color represents a subpopulation component. Each vertical bar represents a sample, with 1–78 being ZS and 79–193 being PT. Population genetic structure analysis assumes that several lines originated from K distinct (or widely differing) ancestors and analyses the proportions of each hypothetical ancestral component each line has in its genetic composition. The best results are obtained when K = 2; **c**: The linkage disequilibrium (LD) decay analysis. PT indicates Plateau type Tibetan sheep, and ZS denotes Zhashijia sheep

Based on SNPs, a phylogenetic tree was inferred using the Maximum Likelihood (ML) method and revealed a paraphyletic relationship between the PT and ZS populations (Supplementary Fig. 2). In line with the phylogenetic tree, STRUCTURE analysis indicated that when K equaled 2, PT and ZS were not completely genetically distinguished (Fig. 1b and Supplementary Fig. 3). Component Analysis (PCA) showed that PC1 and PC2 accounted for 1.15% and 0.97% of differences, and PT and ZS were separated into two groups along the PC1 axis (Fig. 1a). The decay of linkage disequilibrium (LD) demonstrated that PT had a higher LD rate than ZS, implying greater genetic diversity in the former and higher selection pressure on the latter population (Fig. 1c). These observations indicated that the artificial selection led to the genetic differentiation between PT and ZS, suggesting the incorporation of population stratification as a covariate in the following GWAS to minimize confounding effects.

### Genome-wide association studies of body size traits

The IBS-based kinship matrix indicated that most individuals are unrelated, and some are genetically related in 193 Tibetan sheep (Supplementary Fig. 4), suggesting the consideration of kinship in GWAS. Additionally, we included sex and age as fixed effects to mitigate their influence on specific phenotypes, considering that these variables generally influence body size values.

Based on the criterion of a -log_10_ (P-value) more significant than 5, BLINK, GLM, MLM, and SUPER models identified 520, 287, 166, and 398 SNPs significantly correlated with body size traits. The four algorithms existed to detect the same SNP loci, so we integrated and analyzed the number of loci detected by each of the four algorithms. It was determined that the four algorithms concurrently identified a total of 623 SNP loci associated with body size traits, including 98 SNPs for body weight, 35 SNPs for body height, 116 SNPs for body length, 41 SNPs for chest circumference, 87 SNPs for chest depth, 56 SNPs for chest width, and 40 SNPs for pipe circumference as well as an additional set of 150 SNPs related to waist angle width.

**Fig. 2:**
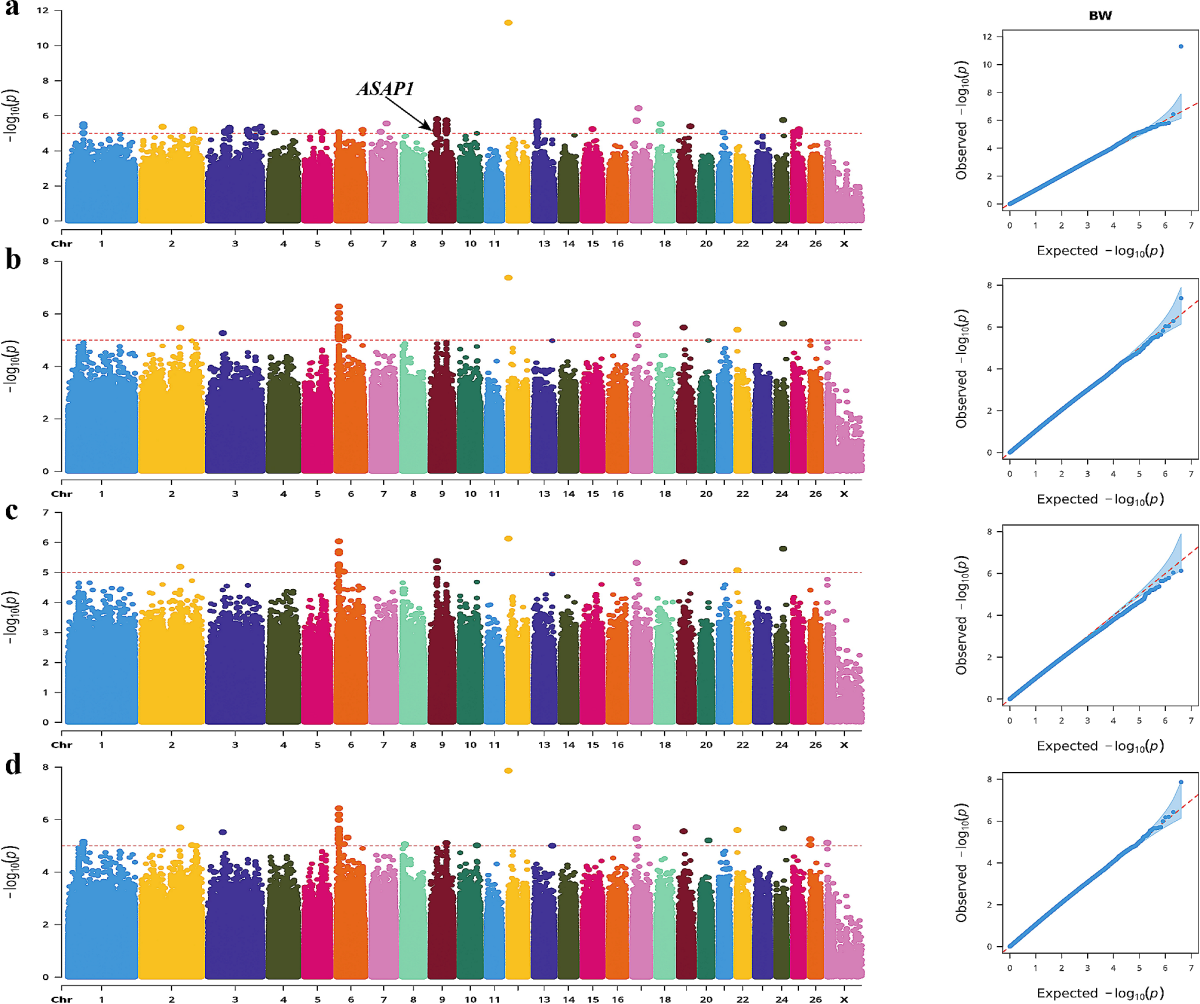
Manhattan and QQ plots of BW trait. (**a**). BLINK; **(b)** GLM **(c)** MLM **(d)** SUPER. The horizontal coordinates are all chromosomes, and the vertical coordinates are loci -log (P-value); four analysis models were used (**a**, **b**, **c**, and **d**) for GWAS analysis, the red dashed line is the threshold for -log_10_ (P-value), and points beyond the threshold range are significantly associated loci

After gene annotation, 10 candidate genes associated with body size traits were ultimately identified, among which 16 SNP mutations were detected. Of these, 98 variants exhibited significant correlations with body weight (see Table S7 and Fig. 2 for detailed information). These body weight-correlated SNPs were annotated in 20 genes, and the most statistically significant SNP (Chr9: 23,424,482 G > A mutation) occurred in the intron of ArfGAP with SH3 domain, ankyrin repeat and PH domain 1 (*ASAP1*) gene.

**Fig. 3:**
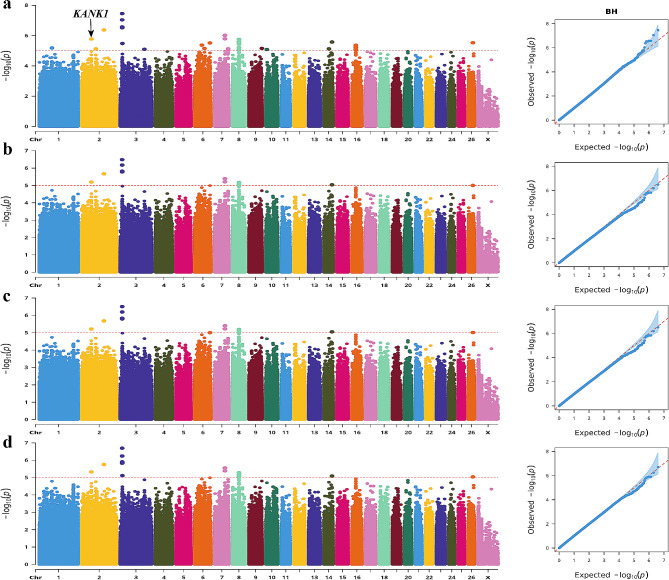
Manhattan and QQ plots of BH trait. **a**. BLINK; **b**. GLM **c.** MLM **d**. SUPER. The horizontal coordinates are all chromosomes, and the vertical coordinates are loci -log_10_ (P-value); four analysis models were used (**a**, **b**, **c**, and **d**) for GWAS analysis, the red dashed line is the threshold for -log_10_ (P-value), and points beyond the threshold range are significantly associated loci

The BH trait was closely associated with 35 SNPs (Fig. 3). Annotation revealed one SNP-annotated gene linked to the body size traits. This specific SNP (Chr2: 69,002,239 C > G mutation) was in the intron of the KN motif and ankyrin repeat domains 1 (*KANK1*) gene.

The Manhattan plot revealed significant associations between 41 SNPs and the CC trait, annotated in 11 genes (Fig. 5). Notably, the most significant SNP within neuron navigator 2 (*NAV2*) gene was identified on Chr21 as a T > G mutation (position: 22,788,649) occurring in an intron. Additionally, one significant SNP (Chr2: 34,862,294 G > A mutation) was annotated in the intron of neurotrophic receptor tyrosine kinase 2 (*NTRK2*) gene. Furthermore, five SNPs were found within the genomic interval of 39,525,865 − 39,557,828 on chr19. These five mutations (C > T, T > A, G > T, A > G and A > G) are all located in the intronic region of protein tyrosine phosphatase receptor type G (*PTPRG*) gene.

Statistically, 87 single nucleotide polymorphisms (SNPs) reached genome-wide significance for the CD trait (Fig. 7), and four genes associated with body size traits were identified through gene annotation. Among them, one significant SNP (Chr4: 10,696,280 C > T mutation at the intron) was located within the intron region of cyclin dependent kinase 6 (*CDK6*). Additionally, two significant SNPs (Chr6: 68,113,923 A > G mutation at the intron; Chr9: 23,072,987 T > C mutation at the intron) were annotated as the FRY like transcription coactivator (*FRYL*) and adenylate cyclase 8 (*ADCY8*) genes, respectively. Furthermore, a significant SNP site on chr10 was observed to have a G > A mutation (position: 68,522,087) within its intron region, which was annotated as the glypican 6 (*GPC6*) gene.

According to the Manhattan plot, 150 SNPs were identified as significantly associated with the WW phenotype (Fig. 8). These SNPs were annotated within a set of 21 genes. *CDK6* and protein tyrosine phosphatase receptor type M (*PTPRM*) emerged as potential candidates influencing body size traits among these genes. Notably, a C > T mutation was observed at position 41,029,246 on chromosome 23, representing a significant SNP site within the *PTPRM* gene’s intron region.

**Fig. 4:**
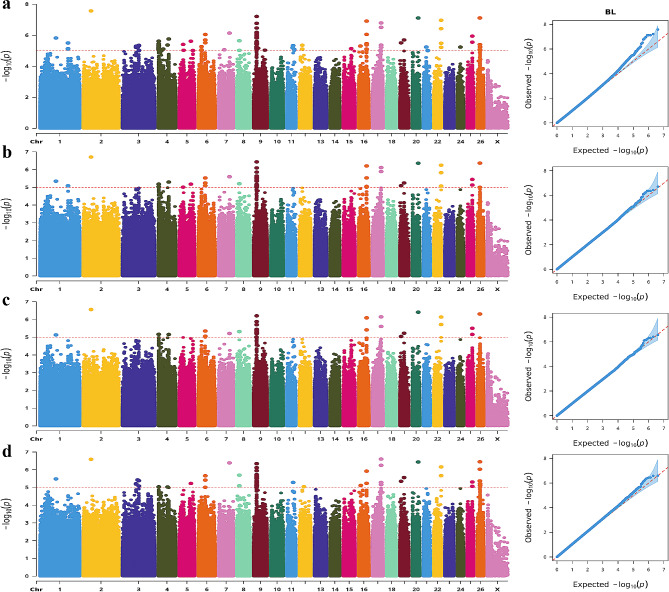
Manhattan and QQ plots of BL trait.**a**. BLINK; **b**. GLM **c**. MLM **d.** SUPER. The horizontal coordinates are all chromosomes, and the vertical coordinates are loci -log_10_ (P-value); four analysis models were used (**a**, **b**, **c**, and **d**) for GWAS analysis, the red dashed line is the threshold for -log_10_ (P-value), and points beyond the threshold range are significantly associated loci

This study’s BL trait was significantly associated with 116 SNPs in 17 genes (Fig. 4). Besides, the CW phenotype demonstrated an association with 56 SNPs that were annotated to 15 genes (Fig. 6). The combined analysis revealed that 40 SNPs, annotated to seven specific genes, were significantly associated with PC traits (Fig. 9). Among these SNP-annotated genes, no genes were found to be associated with body size traits.

The QQ plots revealed a consistent relationship between most loci’s actual and theoretical values, indicating the effectiveness of the four methods in hierarchical population control.

**Fig. 5:**
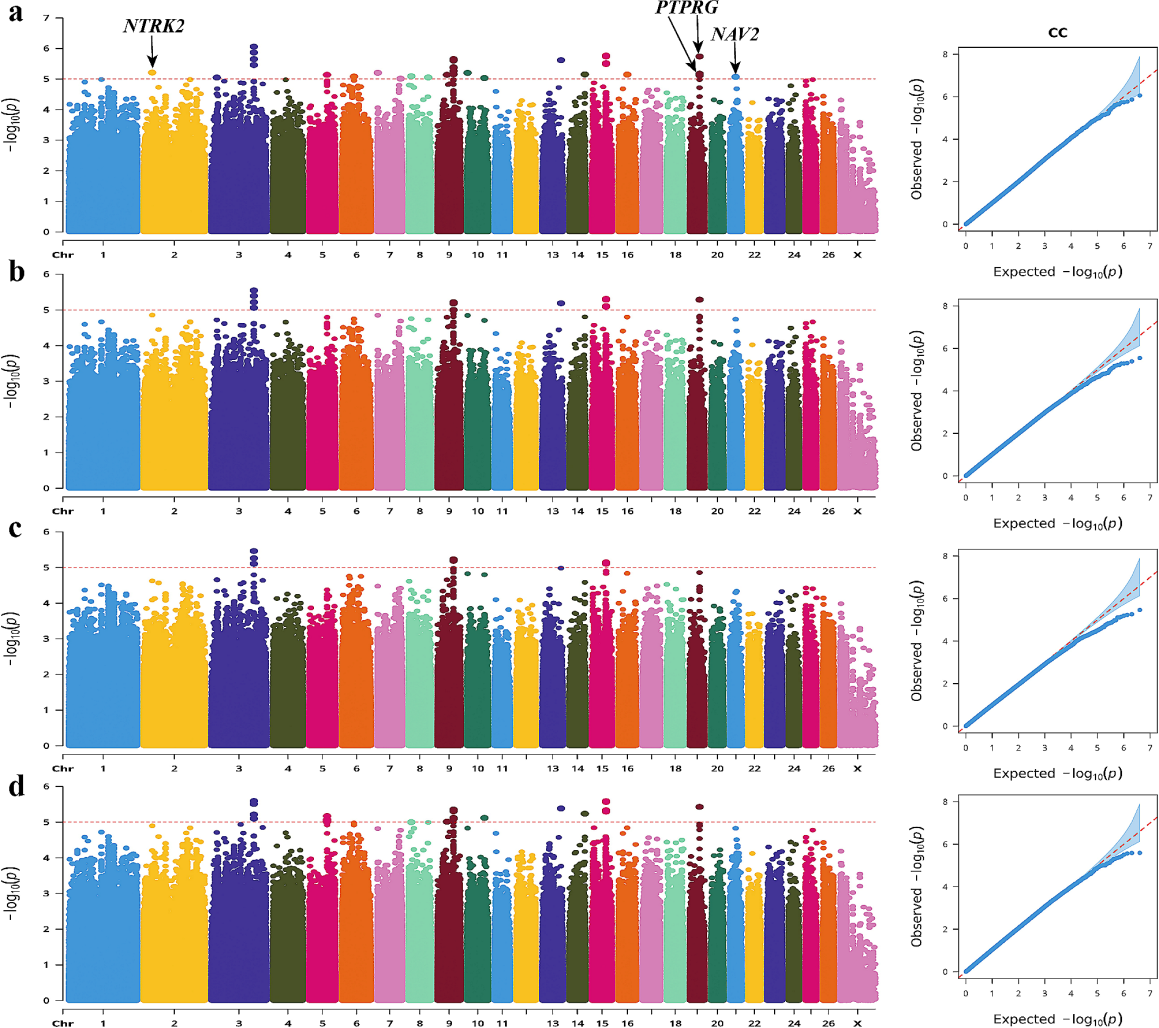
Manhattan and QQ plots of CC trait. **a**. BLINK; **b**. GLM **c**. MLM **d.** SUPER. The horizontal coordinates are all chromosomes, and the vertical coordinates are loci -log_10_ (P-value); four analysis models were used (**a**, **b**, **c**, and **d**) for GWAS analysis, the red dashed line is the threshold for -log_10_ (P-value), and points beyond the threshold range are significantly associated loci

### Enrichment analysis

GO and KEGG enrichment analyses were performed to comprehensively assess the function of candidate genes annotated by SNPs (Supplementary Table S8 and Fig. 10). The eight phenotypes (BW, BH, BL, CC, CD, CW, WW, and PC) were collectively submitted for enrichment analysis. Through GO enrichment analysis, we identified a total of 10 significantly related terms, with genes mainly distributed in the terms “cytoskeleton”, “cytoskeletal protein binding”, “actin filament-based processes”, and “nervous system development”. The KEGG enrichment analysis revealed significant gene enrichment in signal transduction and metabolism pathways, including the cAMP and Rap1 signaling pathways, bile secretion, and Insulin secretion. Candidate gene *ASAP1*, *CDK6*, *FRYL*, *NAV2*, *PTPRM*, *GPC6*, *PTPRG*, *KANK1*, *NTRK2*, and *ADCY8* were identified to be involved in these function pathways and go terms, suggesting their roles in controlling body size through the regulation of metabolism.

**Fig. 6:**
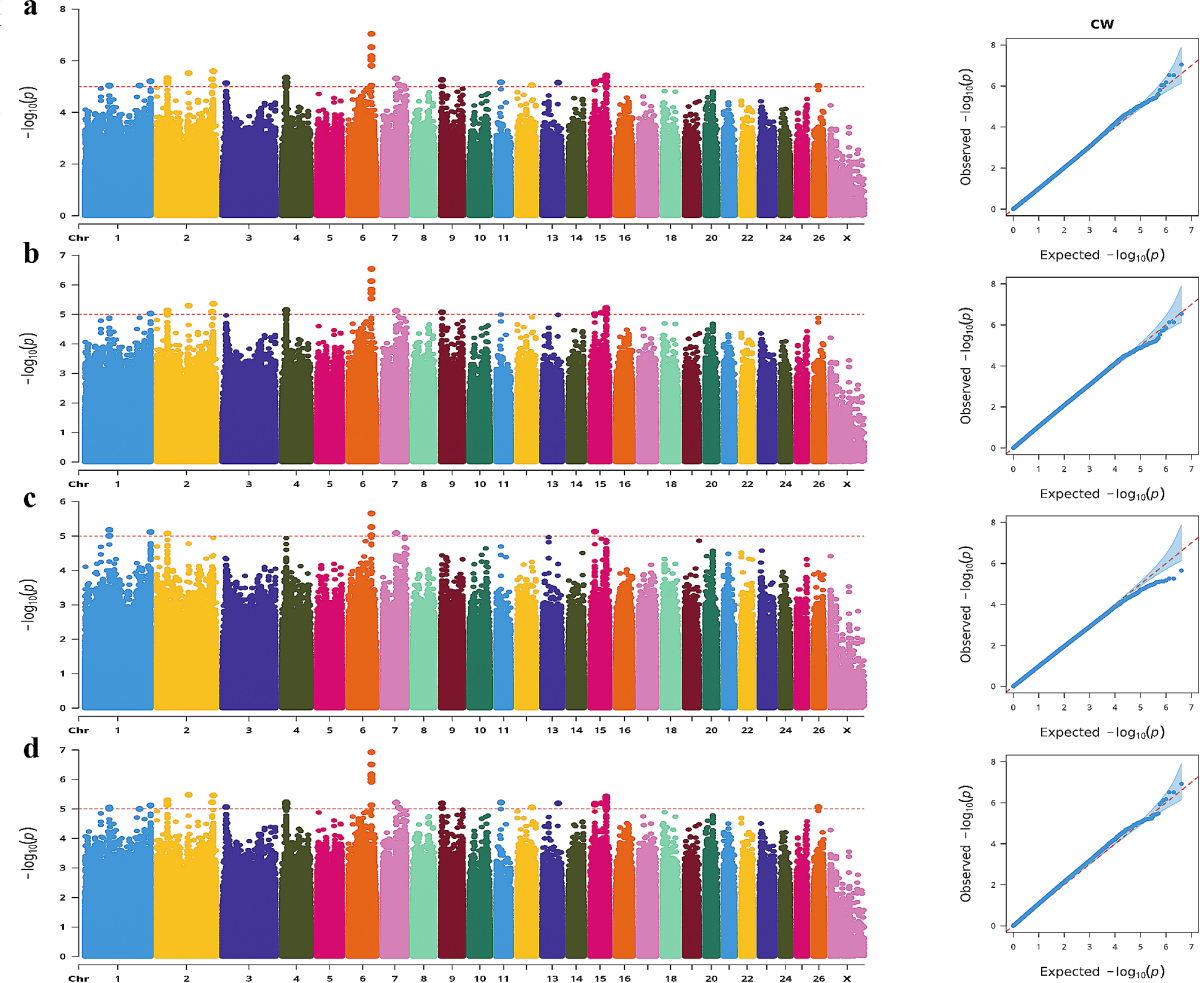
Manhattan and QQ plots of CW trait. **a**. BLINK; **b.** GLM **c**. MLM **d**. SUPER. The horizontal coordinates are all chromosomes, and the vertical coordinates are loci -log_10_ (P-value); four analysis models were used (**a**, **b**, **c**, and **d**) for GWAS analysis, the red dashed line is the threshold for -log_10_ (P-value), and points beyond the threshold range are significantly associated loci

**Fig. 7:**
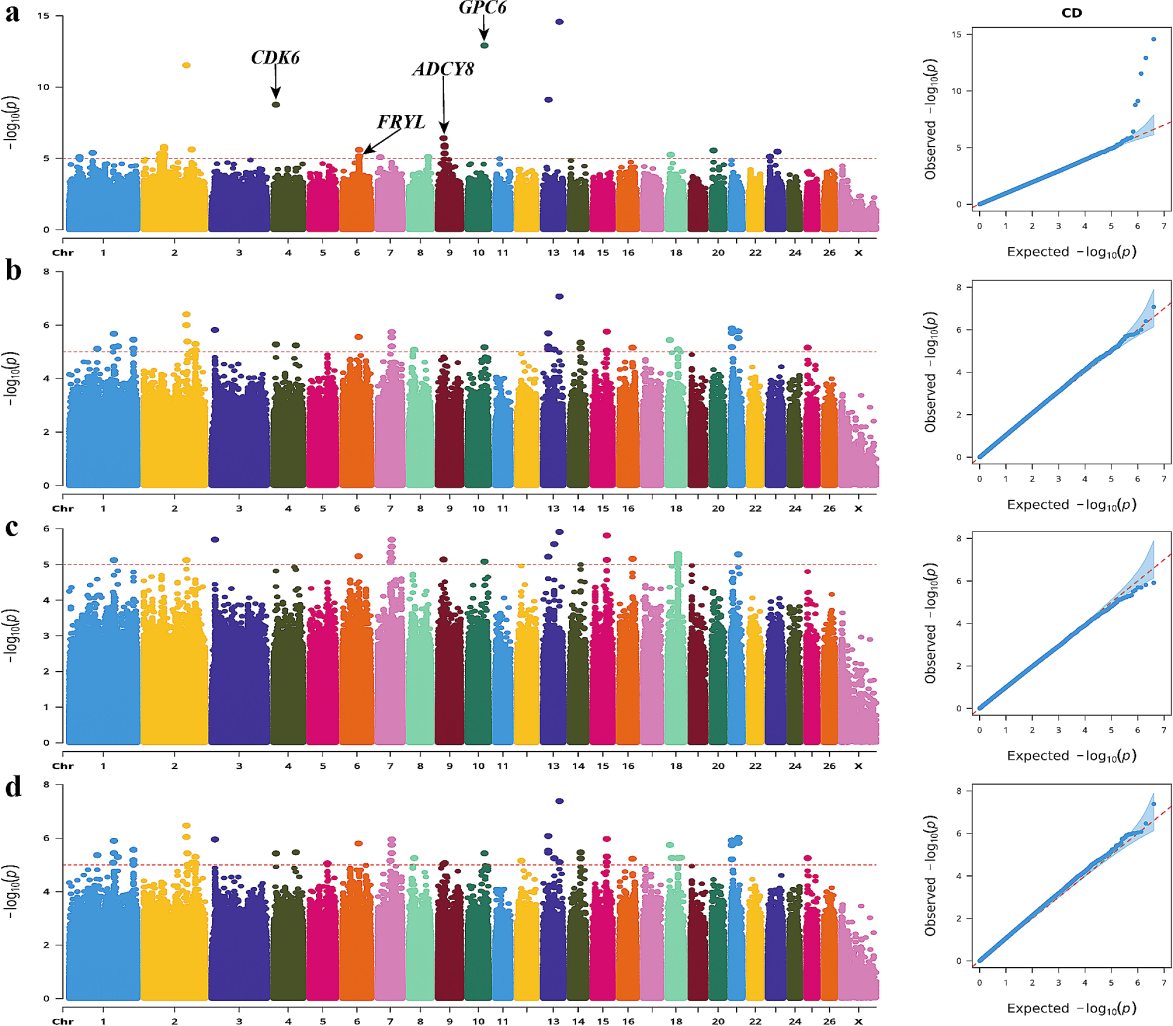
Manhattan and QQ plots of CD trait. **a.** BLINK; **b**. GLM **c**. MLM **d**. SUPER. The horizontal coordinates are all chromosomes, and the vertical coordinates are loci -log_10_ (P-value); four analysis models were used (**a**, **b**, **c**, and **d**) for GWAS analysis, the red dashed line is the threshold for -log_10_ (P-value), and points beyond the threshold range are significantly associated loci

## Discussion

Compared to commercial and domestic superior breeds, Tibetan sheep exhibit disadvantages in body size and growth rate. Body size encompasses various intricate traits such as body weight, height, length, chest depth, width, circumference of the chest and pipe, and waist angle width. Enhancing these traits through traditional breeding methods within a limited timeframe proves challenging due to slow progress rates, low selective accuracy for individual characteristics, high input costs, and time-consuming processes. With the rapid advancements in sequencing and statistical methods, genetic markers and candidate genes were detected to be associated with body size traits, facilitating the breeding progression. In this study, we conducted genome-wide sequencing and association studies to identify SNPs and candidate genes influencing body size in the Tibetan sheep (PT and ZS breeds), which lays the foundations for the genetic enhancement of growth performance in sheep breeding.

In correlation analyses, group stratification and individual kinship are commonly recognized as the primary factors that affect false-positive associations. To mitigate these influences, the leading analytical tool, GAPIT, with multiple algorithms (GLM, MLM, CMLM, ECMLM, SUPER, FarmCPU, and BLINK), was adopted for genome-wide association analysis in the current study [23, 24].

Currently, GWAS studies have primarily focused on reproductive and meat quality traits [25, 26], with limited exploration of body size traits in sheep, such as Hulunbeier and Lake sheep. In contrast, Tibetan sheep have garnered more attention for their environmental adaptation capabilities. Therefore, we conducted this study to address this research gap. Zhang et al. discovered a significant association between OAR17_14085599 SNPs and chest circumference in Hulun Buir sheep [27]. Jiang et al. identified five SNPs associated with body height and four SNPs associated with chest circumference in Hu sheep [7]. In our study, we detected 16 noteworthy SNPs at the genomic level: one linked to BW, one related to BH, seven linked to CC, four connected to CD, and three linked to WW. These SNPs are located within intronic regions, which might impact body size traits by regulating the splicing pattern or transcription efficiency. Therefore, we conducted a more comprehensive investigation of these SNP mutation sites and performed genomic annotation on these 16 SNPs. As a result, we identified ten potential candidate functional genes that may exert pivotal roles in the growth, development, and regulation of body size.

**Fig. 8:**
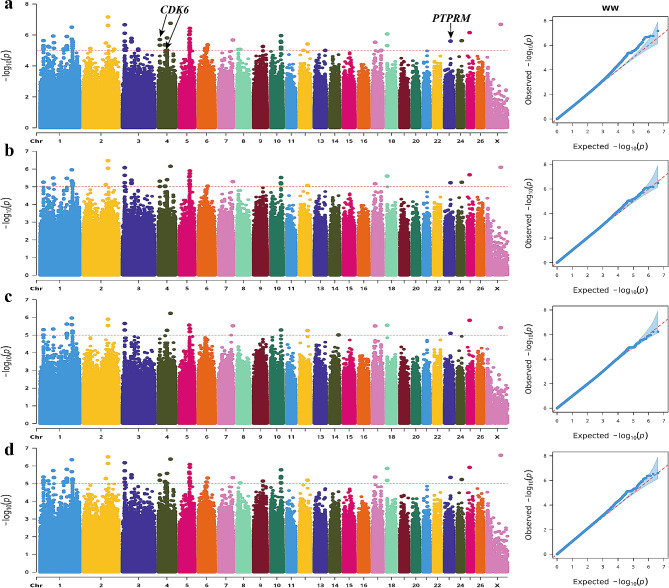
Manhattan and QQ plots of WW trait. **a**. BLINK; **b**. GLM **c**. MLM **d**. SUPER. The horizontal coordinates are all chromosomes, and the vertical coordinates are loci -log_10_ (P-value); four analysis models were used (**a**, **b**, **c**, and **d**) for GWAS analysis, the red dashed line is the threshold for -log_10_ (P-value), and points beyond the threshold range are significantly associated loci.

**Fig. 9:**
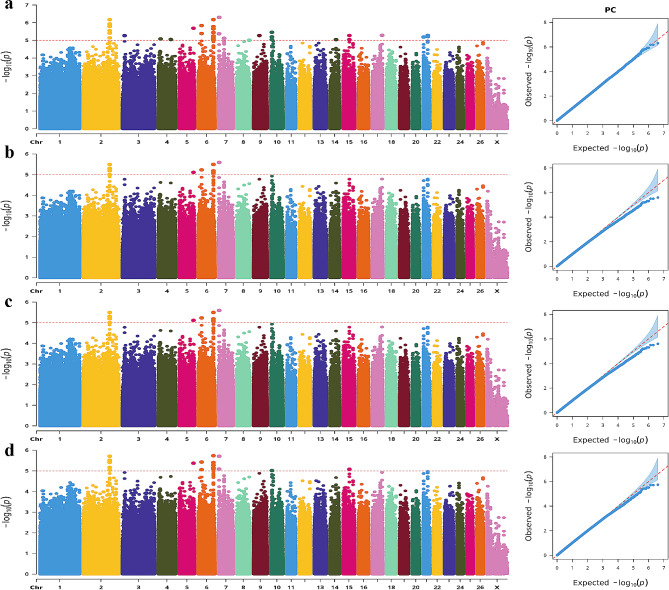
Manhattan and QQ plots of PC trait. **a**. BLINK; **b**. GLM **c**. MLM **d**. SUPER. The horizontal coordinates are all chromosomes, and the vertical coordinates are loci -log_10_ (P-value); four analysis models were used (**a**, **b**, **c**, and **d**) for GWAS analysis, the red dashed line is the threshold for -log_10_ (P-value), and points beyond the threshold range are significantly associated loci

**Fig. 10:**
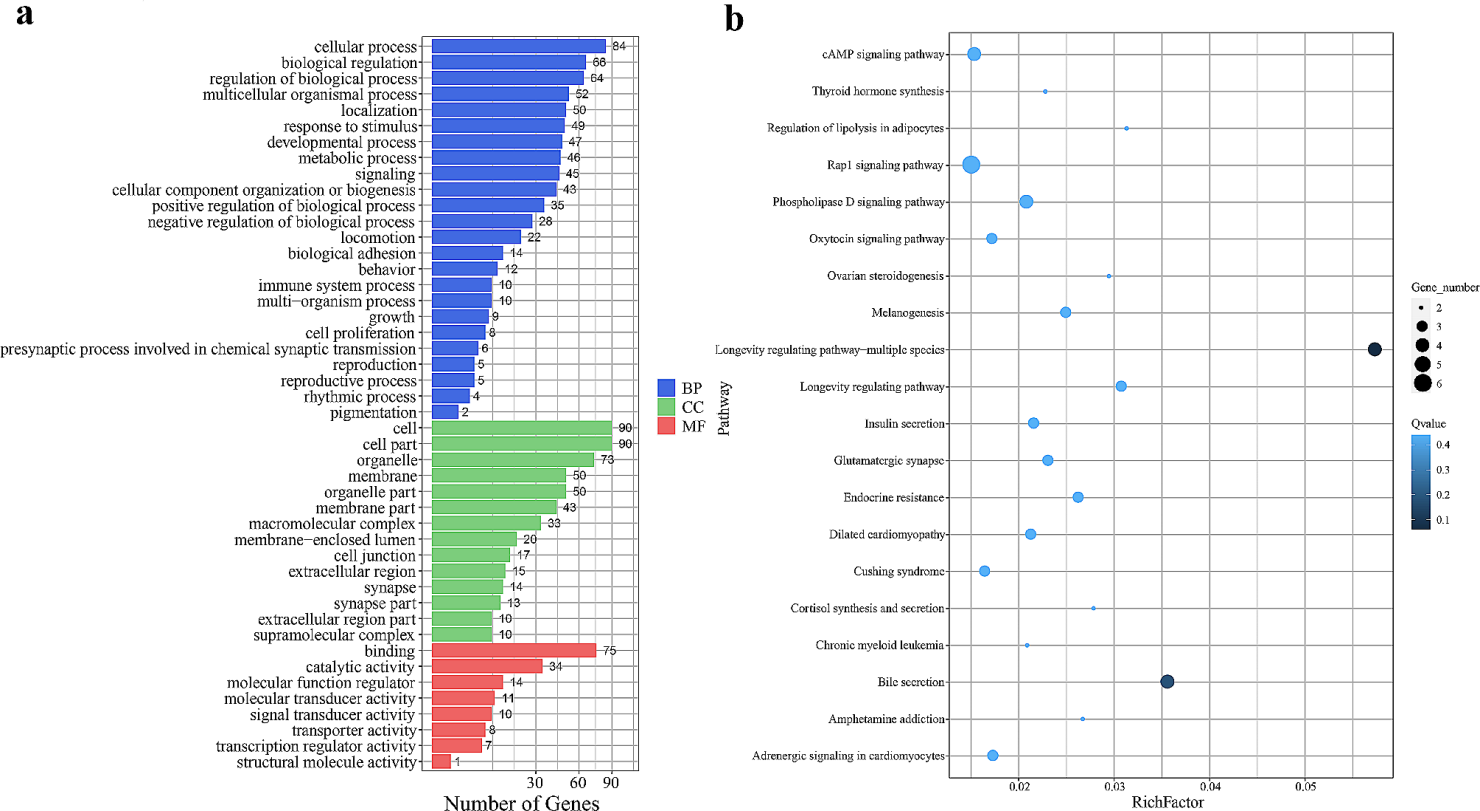
GO and KEGG enrichment analysis of genes in regions of significant association. **a**: GO enrichment analysis; **b**: Bubble chart of the top 20 pathways in KEGG analysis

This study identified SNPs associated with body weight trait and revealed significant associations between the *ASAP1* gene and growth. Consistent with our result, SNPs in the *ASAP1* gene have been reported to be associated with production and growth traits in cattle [28, 29]. It is also linked to growth, adipocyte differentiation, and the formation of fat accumulation in mice [30]. Furthermore, the *ASAP1* gene has also been identified as a candidate gene for age-specific body weights and plays a crucial role in regulating duck growth rate, growth, and development [31]. After conducting an association analysis of CD trait, we successfully identified the genes *FRYL*, *ADCY8*, and *GPC6*. *FRYL* has been suggested as a potential candidate gene influencing the body weight of broiler chickens, thus indicating its significance in chicken growth [32]. Notably, the *ADCY8* gene not only exhibits a strong association with human obesity but also enhances carcass quality and meat quality in sheep [33, 34]. Carcass weight is a body weight characteristic. Enrichment analysis revealed that the *ADCY8* gene was significantly enriched in both the cAMP and Rap1 pathways. The cAMP pathway plays a crucial role in regulating adipogenesis, lipid partitioning in white adipose tissue, as well as lipid metabolism in the liver. Additionally, it is involved in central physiological processes such as cell growth, differentiation, gene transcription, protein expression, and metabolism [35–37]. On the other hand, Rap1 signaling has been shown to have a impact on the β-adrenergic signaling pathway and plays an important role in skeletal muscle growth and development, which are essential for animal growth [38, 39]. These findings further support the possibility that the mutation in *ADCY8* gene may influence body size in Tibetan sheep. The *GPC6* gene has been investigated in mice and pigs, revealing that loss-of-function mutations in this gene lead to reduced stature and shortened limbs in mice [40]. Furthermore, the *GPC6* gene is considered a potential contributor to intramuscular fat deposition in Duroc pigs while exhibiting a significant correlation with the large body size of Sujiang pigs. Additionally, the *GPC6* haplotype plays a role in enhancing the growth performance of Laiwu pigs [41, 42]. The *CDK6* gene was identified to associate with WW and CD traits in Tibetan sheep. A previous study has showed that SNPs in the *CDK6* gene had associations with body length, loin height, and chest circumference in cattle [43]. In addition to domestic animals, this gene has been identified to be associated with body height and morphological dimensions in humans, suggesting its convergent rwuole in regulating body size [44]. Additionally, the WW trait association analysis revealed the involvement of the *PTPRM* gene, which governs a diverse array of cellular processes encompassing cell growth, differentiation, and signaling cascades. It has been reported that genes involved in cell growth and differentiation experienced selection during domestication and might contribute to an enhanced growth rate in pigs [45]. CC trait association analyses identified the *PTPRG* gene as a potential QTL gene for growth and linear traits [46]. *NAV2* gene exhibited a significant association with mouse height [47]. The genes *GPC6*, *CDK6*, *PTPRM*, *PTPRG*, and *NAV2* have been found to influence body size in various animal species. Therefore, we postulated that these genes might be correlated with phenotypic traits related to body size in Tibetan sheep.

In the current study, we newly identified the significant association between *NTRK2* and *KANK1* genes with CC and BH traits in Tibetan sheep. The *NTRK2* gene regulates human obesity [48, 49]. At the same time, Kank1 has been demonstrated to play a crucial role in the actin cytoskeleton dynamics and is commonly expressed across mammalian tissues. Moreover, actin cytoskeleton dynamics exert significant regulatory control over muscle development, growth, and regeneration by governing fundamental processes such as cell proliferation, differentiation, and morphological changes [50]. Therefore, *NTRK2* and *KANK1* genes may influence body size traits in Tibetan sheep and serve as potential candidates for genetic selection.

Identifying SNPs and candidate genes associated with body size traits will provide valuable resources for enhancing growth performance in Tibetan sheep. In this study, we have identified ten genes (*ASAP1*, *CDK6*, *FRYL*, *NAV2*, *PTPRM*, *GPC6*, *PTPRG*, *KANK1*, *NTRK2*, and *ADCY8*) potentially influencing the phenotypic variation in body size of Tibetan sheep. Subsequently, we aim to investigate their transcriptional and protein expression levels to discern any differential regulation and explore the enriched pathways associated with these genes to elucidate the underlying mechanisms governing body size regulation in Tibetan sheep.

## Conclusion

Conclusively, we conducted genome-wide sequencing of 193 sheep from two Tibetan sheep breeds (PT and ZS) and performed GWAS using four models to investigate eight phenotypes related to body size. In total, 623 SNPs were significantly associated with eight body size traits. Based on these SNPs, 111 candidate genes were identified. Enrichment analysis revealed that *ASAP1*, *CDK6*, *FRYL*, *NAV2*, *PTPRM*, *GPC6*, *PTPRG*, *KANK1*, *NTRK2*, and *ADCY8* genes were involved in pathways and terms related to growth and development, which might contribute to the regulation of the body size. Considering the findings above collectively, these genes may play crucial roles in determining body size traits in Tibetan sheep. The present study has successfully identified many significant SNPs associated with body size traits in sheep, thereby establishing a robust theoretical foundation for enhancing sheep production performance. Further analyses are required to characterize the functions of these genes to resolve the genetic mechanisms affecting sheep phenotypes.

## Materials and methods

### Animal ethics

The animal-related procedures were strictly conducted by the guidelines and regulations established by the Institutional Animal Care and Use Committee of the Northwest Institute of Plateau Biology, Chinese Academy of Sciences (Approval No. NWIPB2023015). The animal owners provided informed consent to include all animals in the study.

### Sample collection and phenotypic recording

The ear tip tissues were collected from 193 Tibetan sheep (1 to 3.5 years), comprising 115 plateau-type sheep (56 rams and 59 ewes) and 78 Zhashijia Tibetan sheep (20 rams and 58 ewes). These samples were obtained from the Qinghai Sheep Breeding and Promotion Service Center in Gangcha County and a cooperative in Qumalai County, Qinghai Province, China. The ear tip tissues were preserved in 75% alcohol at -20 °C for subsequent DNA extraction. Eight phenotypes of body size traits were recorded in 193 Tibetan sheep, including body weight, body length, body height, chest circumference, chest depth, chest width, waist angle width, and pipe circumference. We utilized the PASW Statistics 18 software to establish correlations among the eight phenotypic traits.

### Whole-genome sequencing

Genomic DNA was extracted and purified from 193 Tibetan sheep samples using a tissue kit (BGI-Shenzhen, China). DNA of 0.5 µg was used for library construction based on the manufacturer’s description, with an insert size of approximately 300 bp. After PCR and purification, the sequencing library was sequenced in the DNBSEQ platform (BGI-Shenzhen, China).

We employed the software SOAPnuke (version: 2.2.1) [51] for quality control and filtration of the raw data. Subsequently, BWA’s mem algorithm (version: 0.7.17-r1188) [52] was utilized to align clean reads against the reference genome (GCF_016772045.1_ARS-UI_Ramb_v2.0), which was then converted into a sorted bam file using samtools (version: 1.9) [53]. Finally, we performed quality control statistics on the resulting bam file using Qualimap2’s bamqc tool (version: 2.2.2-dev) software [54]. GATK (version: 4.2.6.1) was employed for SNP calling with default parameters [55], followed by SNP annotation using SnpEff [56].

### Population genetics analyses

The ML phylogenetic trees for all samples were constructed using FastTree software (version: 2.1.11) [57], and the resulting tree construction outcomes were visualized with the R package ggtreee [58].

Structure analysis of all samples was performed using admixture (version: 1.3.0) [59] as follows:

Firstly, PLINK (version: 1.07) software [60] was used to convert the VCF file into the input file required by the admixture software; Second, several K-values were set to analyze the proportion of each hypothetical ancestor component in the genetic composition of each sample; Finally, the cross-validation errors of the individual results were compared, and the K-value with the lowest error rate was selected as the optimal result for the genetic structure. The structure corresponding to the K-value with the lowest error rate was chosen as the optimal result.

GAPIT3 (version: 3.2) in R [25] was used for Principal Component Analysis (PCA) and Kinship Analysis (KA) on all samples. For PCA, we presented the clustering results of all models based on the first two principal components (PC1 and PC2). Regarding KA, we utilized the VanRaden algorithm to calculate kinship matrices among all samples. Linkage disequilibrium (LD) decay was performed using PopLDdecay (version: 3.41) to calculate the degree of LD coefficient (r^2^) for pairwise SNPs [61].

### Genome-wide association study (GWAS)

Four algorithms were applied for GWAS analysis, including General Linear Model (GLM) [62, 63], Mixed Linear Model (MLM) [64], Settlement of MLM Under Progressively Exclusive Relationship (SUPER) [65], and Bayesian-information and Linkage-disequilibrium Iteratively Nested Keyway (BLINK) [66] modeling. The SNPs in this study were filtered using the vcftools software (Version: 0.1.16) with the following parameters: a minor allele frequency (MAF) threshold of 0.05, a maximum allowable missing value of 1, a minimum mean depth requirement (meanDP) of 10, and a minimum genotype quality (GQ) threshold of 10. Subsequently, after setting fixed thresholds, SNPs exhibiting -log_10_(P-value) greater than five were considered statistically significant associations [67–69]. The CMplot (version: 4.4.1) was applied to generate Manhattan and Quantile-quantile plots to visualize GWAS results [70]. The formula of the GAPIT3 software is presented below:

$${\rm{y = X\beta + S\alpha + Qv + Zu + e}}$$

In this model, y represents trait values; X is environmental effect (place and time of phenotype measurement, etc.); S signifies a fixed effect of SNP loci; Q is PCA, age, and gender fixed effect; Z indicates the Kinship matrix; e stands for Error: random effect.

### Identification of candidate genes and enrichment analysis

Based on LD attenuation distance analysis, we investigated candidate genes’ biochemical function at the significant SNP locus Physical location. The sheep reference genome (ARS-UI_Ramb_v2.0; GCF_016772045.1) was used to annotate these genes. These genes were aligned to the UniProt and KEGG databases using the Blastx tool of the sequence alignment software Diamond. The alignment value was set to 1e-05, and each gene’s GO number and KO number were extracted. The target genes were subjected to GO (http://www.geneontology.org/) and KEGG Pathway enrichment analyses.

### Electronic supplementary material

Below is the link to the electronic supplementary material.

Supplementary Material 1

Supplementary Material 2

Supplementary Material 3

Supplementary Material 4

Supplementary Material 5

Supplementary Material 6

Supplementary Material 7

Supplementary Material 8

Supplementary Material 9

Supplementary Material 10

Supplementary Material 11

Supplementary Material 12

## Data Availability

The sequencing datasets were deposited in NCBI SRA under PRJNA1111723. The phenotype data of body size traits can be accessed in Table 1 and Table S1.

## Abbreviations

GWAS: Genome-Wide Association Studies
SNP: Single Nucleotide Polymorphism
PT: Plateau type Tibetan sheep
ZS: Zhashijia Sheep
BW: Body Weight
BH: Body Height
BL: Body Length
CC: Chest Circumference
CW: Chest Width
CD: Chest Depth
WW: Waist Angle Width
PC: Pipe Circumference
PCA: Principal Component Analysis
LD: Linkage Disequilibrium
GLM: general Linear Model
MLM: Mixed Linear Model
BLINK: Bayesian-Information and Linkage-Disequilibrium Iteratively Nested Keyway
SUPER: Settlement of MLMs Under Progressively Exclusive Relationship
QQ: Quantile-Quantile
GO: Gene Ontology
KEGG: Kyotoencyclopedia of Genes And Genomes
