# Supplementary figures and images for: Genome-wide association studies of body size traits in Tibetan sheep
Source: BMC Genomics. 2024 Jul 30;25:739. doi: 10.1186/s12864-024-10633-3 (PMC11290296; doi:10.1186/s12864-024-10633-3)

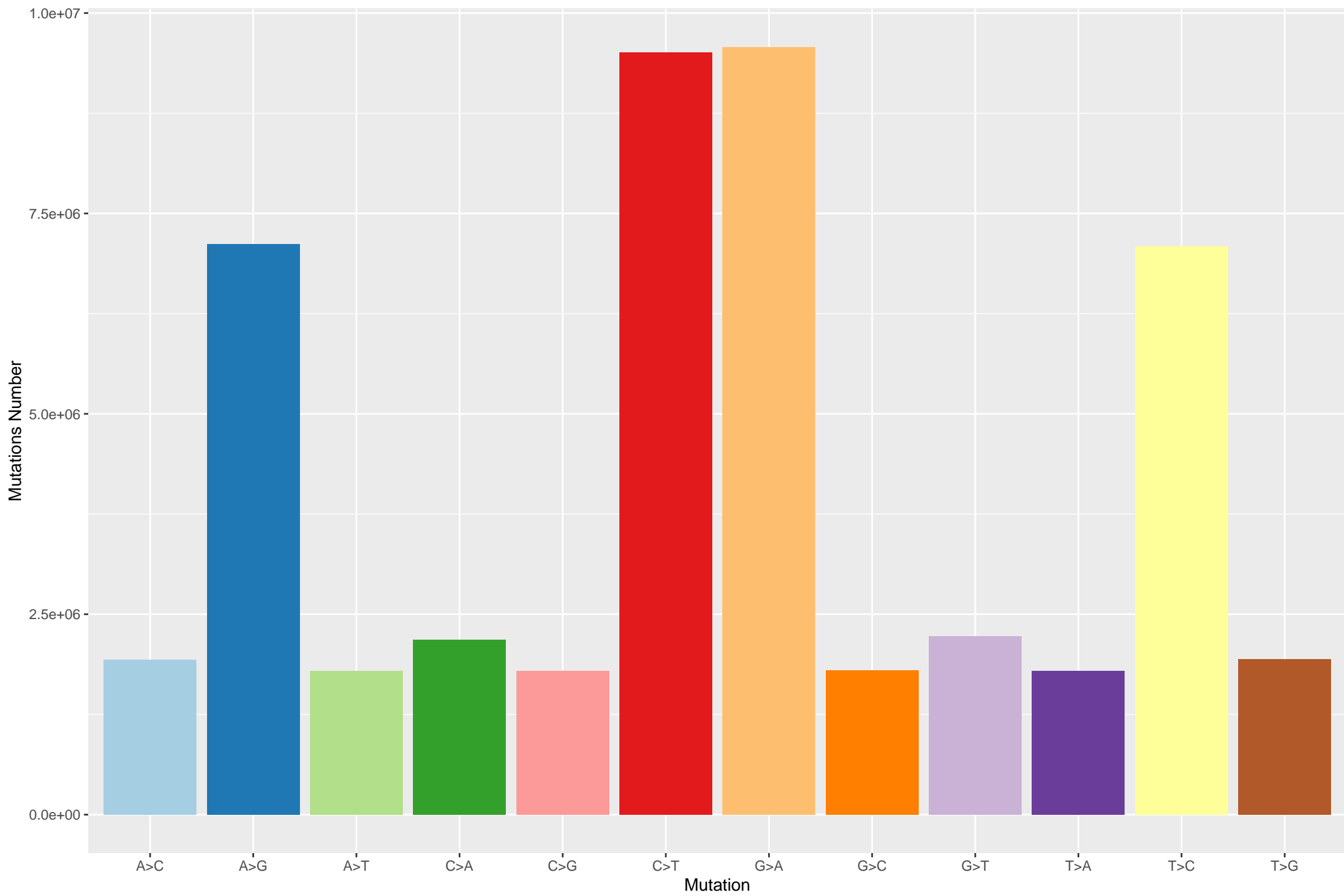

Supplement: Supplementary file 1 — Supplementary Material 1 [file 12864_2024_10633_MOESM1_ESM.pdf]

**Cross validation error**

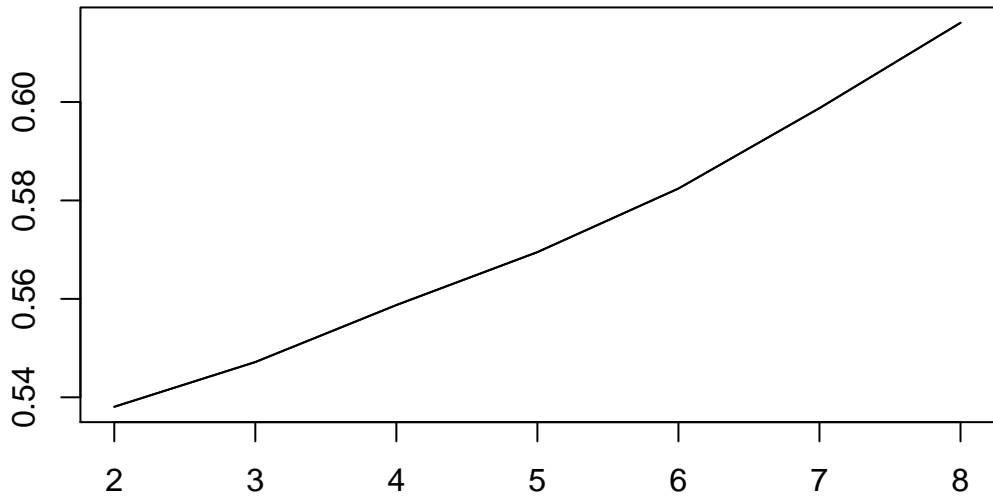

**K-value**

Supplement: Supplementary file 2 — Supplementary Material 2 [file 12864_2024_10633_MOESM2_ESM.pdf]

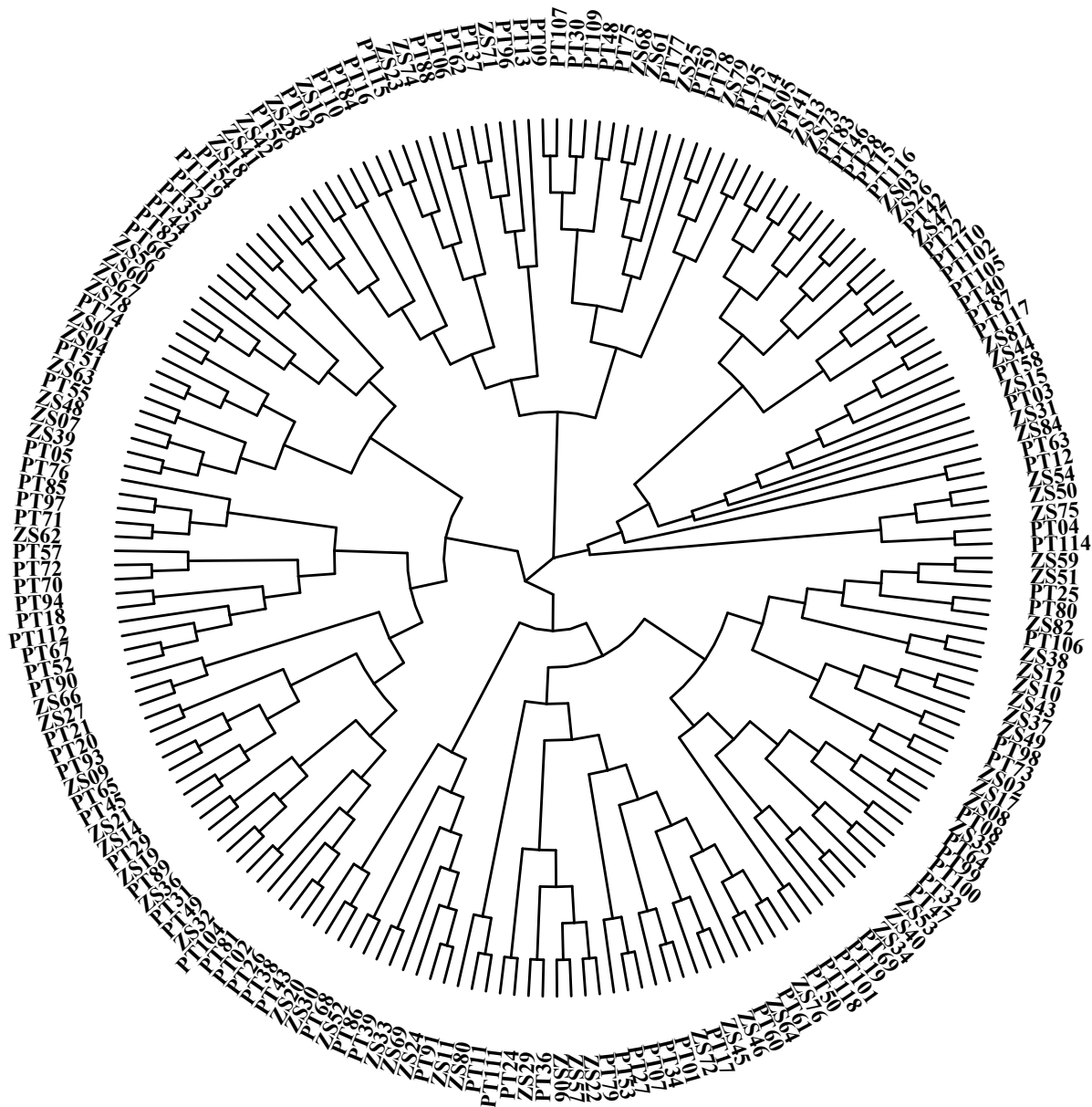

Supplement: Supplementary file 3 — Supplementary Material 3 [file 12864_2024_10633_MOESM3_ESM.pdf]
