# Supplementary material for: Genome-wide association studies of body size traits in Tibetan sheep
Source: BMC Genomics. 2024 Jul 30;25:739. doi: 10.1186/s12864-024-10633-3 (PMC11290296; doi:10.1186/s12864-024-10633-3)
Supplement: Supplementary file 4 — Supplementary Material 4 [file 12864_2024_10633_MOESM4_ESM.pdf]

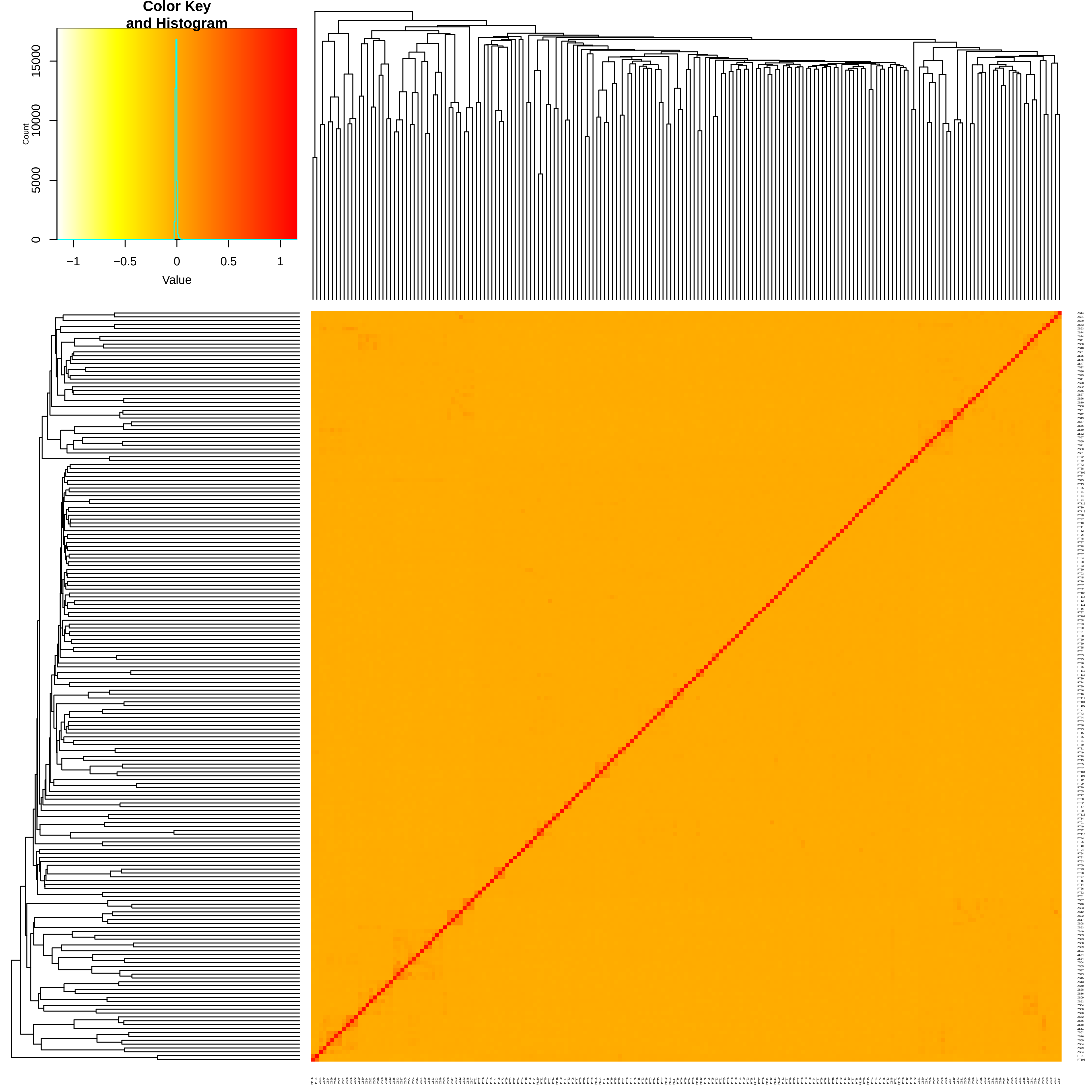
Color Key
and Histogram
Count
0
5000
10000
15000
-1
-0.5
0
0.5
1
Value
